# Anatomo-Functional Correlation between Head Zones and Acupuncture Channels and Points: A Comparative Analysis from the Perspective of Neural Therapy

**DOI:** 10.1155/2014/836392

**Published:** 2014-11-25

**Authors:** Martha Liliana Beltrán Molano, Laura Bibiana Pinilla Bonilla, Eduardo Humberto Beltrán Dussan, Carlos Alberto Vásquez Londoño

**Affiliations:** Faculty of Medicine, Universidad Nacional de Colombia, Bogota, Colombia

## Abstract

*Background*. Neural therapy and traditional Chinese medicine (TCM) are part of complementary and alternative medicine in western world. Both of them share characteristics in diagnosis and therapeutics in search of changes in tenderness, pain, and skin stiffness related to visceral disease, as well as therapeutic procedures with specific stimuli on the skin that generate local, segmental, or remote reactions. Head zones explain segmental viscerocutaneous relations in neural therapy; however, interference fields and remote reactions after infiltration of local anesthetic go beyond this segmental distribution. *Methods*. This descriptive research required review and analysis of texts of Henry Head and traditional Chinese medicine. *Results*. Anatomical and functional relationships were found between Head zones in body, and head and neck with 14 acupuncture channels and their points. Anatomical areas of strong correlations were found: Head zones of heart and lung with heart and pericardium channels; Head zones of genitals with bladder and kidney channels. Strong functional relations between all Head zones, channels, and acupoints were found when following the pattern of segmental dermatomes; 235 acupuncture points were found in concordance.

## 1. Introduction

In the Western world the neural therapy and traditional Chinese medicine (TCM) take part of alternative and complementary medicine, also known as* regulatory therapies,* because it activates the organism compensatory-regulatory mechanisms in order to generate responses against the disease and restore the balance [1].

Neural therapy (or* Neural Therapeutic Medicine* according to Colombian School [2]) is based on the principle of* nervism* where the nervous system acts as a generator and controller shaft of all human biological phenomena [1, 3, 4] and on the basic system according to Pischinger, in which the extracellular space works as a network responsible for organic regulatory processes through interconnections that allow the flow of information throughout the body [1]. Pathological processes or* irritations* are called* interference fields* or* neurodystrophies* that can cause blockages in the basic system or short-circuit in the nervous system leading to imbalance [1, 5, 6]. Diagnosis is based on finding irritations through the clinical history of the patient [6, 7] and the physical examination, based on the segmental theory of Head zones as well as the search of interference fields in scars, teeth, organs, mind, and emotions [1, 6, 7]. The treatment is performed by application of procaine, a local anesthetic with unique chemical and electrical properties [8, 9] which generates local segmental and remote responses in the body.

On the other hand, traditional Chinese medicine has described, for more than 3000 years, an extensive network of channels (*Jin mai*) and collaterals (*Luo mai*) which are channels that transport* qi* (energy) and* xue* (blood). These channels are interconnected forming a network of comprehensive energy communication. Within this network there are 12 main channels and extraordinary vessels as* Ren mai* and* Du mai* which have their own acupuncture points (acupoints) in their paths [10–12]. Each channel is associated with a* zang-fu* organ, in addition to the five senses, tissues, body fluids, and emotions, among others, whose physiological and pathological activity are originally conceived from ancient concepts of yin-yang and the 5 elements rather than from a specific morphology [12, 13]. Therapeutic indications of the points depend on four characteristics: the location of the areas where they are situated, the properties and specific functions of the channel to which they belong, the particular functions of each point, and the* yin* or “*yang*” polarity of the channels [11]. The effect of treatment can be local, segmental, or distal along the channel, including head and neck.

The English neurologist Head (1861–1940) published his doctoral thesis in 1893 in the journal* Brain* [14], which exposes the clinical findings of visceral disease associated with cutaneous manifestations as hyperalgesia or allodynia. Then reviews cases of herpes zoster and discovers similarities with the anatomical distribution of visceral diseases. Sir Head identified corporal segmental zones with defined bounds, later based on Sherrington's research on spinal innervations, creates his map of dermatomes. Head finds two anatomical gaps where there is no cutaneous tenderness of visceral origin, located between cervical levels C5 and C8 and lumbar levels between L2 and L4 [14, 15]. In third part of his work, published in 1894 [16], Head describes skin manifestations of hyperalgesia in head and face and neck associated with local diseases in organs of head and neck and distant diseases in thoracoabdominal viscera, from clinical cases of cephalea.

Sir Head proposes that the tenderness of the face and head, including teeth, does not come from spinal roots but from cranial pairs. As a result of his own findings in research, Head describes zones with defined bounds in head, face, and neck, whose clinical significance has not been previously discussed. Head analyzes the visceral cutaneous findings of Mackenzie (1892) as well as the hypothesis of Ross (1888) and reinforces the concept now known as visceral cutaneous reflexes.

It is currently known that both the nervous system and the epidermis come originally from the ectoderm. Somites are formed from the mesoderm, each one forming its own sclerotome, myotome, and dermatome; the two last ones preserve the innervation of its original segment [17–19]. In face and head, somatesthetic function is carried mostly by the trigeminal nerve [17, 18]. Meanwhile, visceral tenderness is picked up by the enteric plexus and reaches the nuclei in the ganglion neurons of spinal nerve to be connected with the dorsal horn. It is believed that the afferents of dermatomes, myotomes, and visceral nociception make synapse in the Rexed laminae I-II, IV-V of the Gray matter and then through the spinoreticulothalamic pathway they reach the hypothalamus and intralaminar and posterior thalamus, generating skin perception of visceral pain [17–19].

Recent studies have examined the relationship between dermatomes and acupuncture channels and points. Beissner et al. [20] analyzed the points of maximum pain described by Head in each one of his zones comparing them anatomically with* Mu* and* Shu* acupuncture points, finding a clear correlation in lung, stomach, liver, and kidney/ureter Head zones. Cabioglu and Arslan [21] discussed the therapeutic and segmental relationships between* shu* points in the bladder channel and* HuatuoJiaji* points with dermatomes, finding that they keep the same segmental neuroanatomical distribution of dermatomes supporting these findings on the viscerocutaneous reflexes. Ferreira and Luiz [22] statistically analyzed similarities between dermatomes and traditional and contemporary indications of acupuncture points as well as the relationships of these with the nervous system anatomy. Significant associations were found through the variables used.

The purpose of this study is to find the possible anatomical and functional relationships between Head zones and acupuncture channels and points, results that will help in explaining how two different alternative and complementary medical systems supported by their own systems of biological communication have links that can be useful to understand health and disease process in a more comprehensive way. Segmental and remote viscerocutaneous relationships found in this study can be taken into account in the clinical evaluation and performing intervention with procaine in neural therapy.

## 2. Materials and Methods

This is a descriptive research performed in 3 stages: reviews of literature, analysis of information collected comparatively, and illustration of results.

### 2.1. Data Collection

Firstly, data collection was performed through the reading of Head's work: “*On disturbances of sensation with especial reference to the pain of visceral disease*” parts 1, 2, and 3. Secondly, information about the fundamentals of TCM, acupuncture channels, and points in TCM texts was checked.

### 2.2. Analysis of Henry Head's Work

An anatomical comparison between images of Head zones mentioned in his books and the TCM channels and acupoints was performed, indicating the areas of greatest anatomic correlation. Subsequently, clinical cases for each zone, from chapters 3 [14] and 1 [16], were analyzed and also have their respective illustrations. A data table collection was created in order to organize Head's most relevant findings.

Table 1 should be read based on this example: the heart area described by Head correspond to dermatomes D1 to D3; in case of exacerbation of pain, such as angina pectoris, this tender area can extend to D4 to D9 including even cervical area. It is related to distant skin hypersensitivity in head and front nasal, mid orbital, and frontotemporal areas. These findings were derived from those described in his clinical cases of patients with valvular heart disease, myocardial infarction, angina pectoris, and aortic aneurysm.

**Figure 1:**
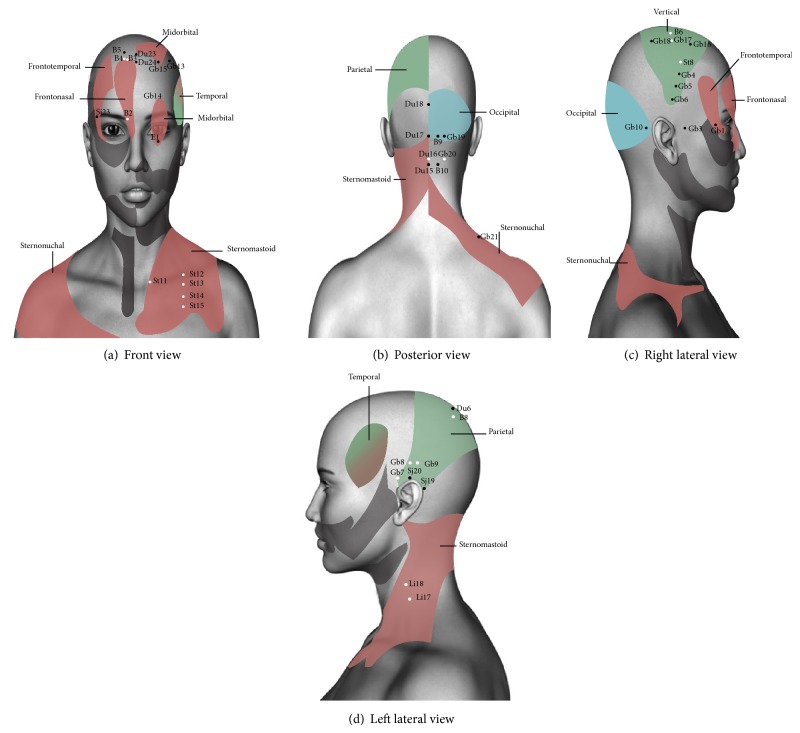
Anatomo-functional correlation in the Head zone of head and neck with acupuncture points with local action (in black) and remote action (in white). The zones are shown in different views.

For easier understanding, head and neck areas are shown in the anatomical figure designed by Head, with the exact zoning (Figure 1); also clinical cases related to them are in the third part of his work [16]. For purposes of this investigation, the analysis focuses on the reflex zones of the head and neck associated with thoracoabdominal visceral pathology.

### 2.3. Analysis of Acupuncture Channels and Points

From the extensive network of acupuncture channels and collaterals, 14 acupuncture channels were selected for this study, including the 12 main channels and Ren Mai and Du Mai, because they have their own acupoints through their paths. Actions of acupuncture points and channels were analyzed according to TCM theory and compared with Head zones activities; these relations in head and neck areas were analyzed according to TCM diagnostic approaches to headache [23].

### 2.4. Integration of Data

Twelve illustrations were designed based on the 3D model developed by Mercado [24], in order to locate Head zones, channels, and acupoints as well as making the anatomical and functional correlation easy to visualize. The comparative process was divided in four corporal segments keeping the distribution of Head zones, in order to facilitate the analysis. The first area comprises the Head zone of head and neck, followed by the upper area that includes the Head zone of heart and lung; the middle area comprises Head zone of stomach, liver, gallbladder, and bowels; and the lower area is made up of kidney, bladder, and genitalia Head zones.

## 3. Results

### 3.1. Head and Neck Zone

In Figure 1 the acupoints with remote and local action are identified, according to the relations found by Head for each area in head and neck [14]. They are also summarized in Table 2.

There are 45 total points related. Table 2 shows that frontonasal, mid-orbital, frontotemporal, temporal, sternonuchal, and sternomastoid zones are related to cardiac and pulmonary function according to Head findings. These areas are also associated with remote pain in corporal segments C3 to D7. At the first four there are 12 acupuncture points with remote and local action. At the frontonasal area B2, B3, and B4 are acupoints associated with fullness-stirring of heart and dyspnea. At sternomastoid and sternonuchal areas of neck there are 12 acupoints with local and remote anatomical and functional correlation, for example, St12 clears heat in chest (see Figure 1 and Table 2) [11, 13, 25].

Temporal, vertical, and parietal areas are associated with remote digestive function and at the same time can be related to pain in corporal segments D7 to D9. At the vertical and parietal zones there are 16 acupuncture points with local and remote action. At vertical zone, St8 shows digestive action, because it relieves vomiting related to headache. At parietal zone there are acupuncture points related to digestive function. For example, Gb8 harmonizes the stomach and alleviates vomiting; B8 point acts on abdominal distension and Sj19 relieves vomiting (see Figure 1 and Table 2) [11, 13, 25]. In TCM temporal area is related to functions of the liver and gallbladder, which are strongly associated with digestive physiology [23].

The occipital zone is associated with digestive and genital functions and the corporal segment D10. No acupoints are found within this area with remote functions; however the occipital headache in TCM is associated with involvement of bladder and kidney channels, meridians related to genitourinary functions of water element [23]. Five acupoints showed local action on neck pain and headache (see Figure 1 and Table 2) [11, 13, 25].

Regarding acupuncture channels, 4 channels with anatomic correlation are observed; the heart channel ends its pathway in infraorbital zone. The stomach channel begins its pathway in infraorbital zone and goes through face; in TCM this channel is related to frontal cephalea [23]. When the liver channel reaches the face it passes through the eye up to the cranial vertex; in TCM the retroocular headache and the upper headache are related to this channel [23]. The gallbladder channel shows an intricate pathway in lateral head and face areas; in TCM lateral and temporal headache is related to this channel [23].

### 3.2. Upper Area (Head Zone of Heart and Lung)

**Figure 2:**
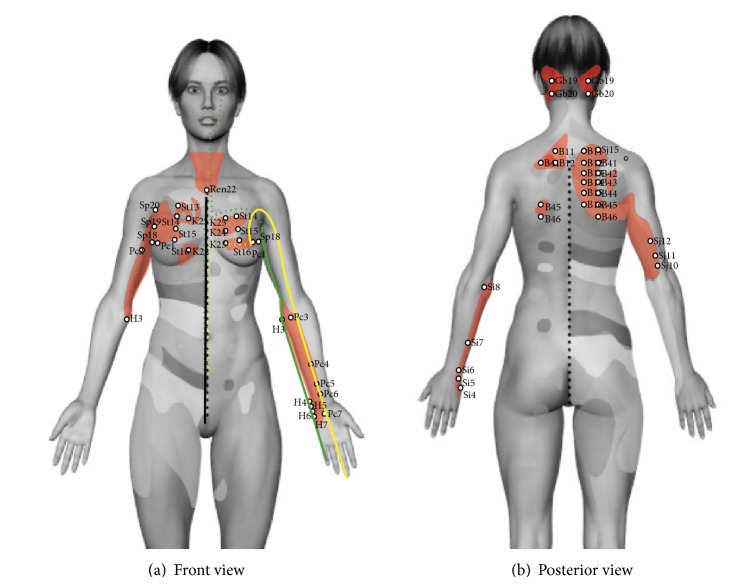
(a) Front view. Heart and lung Head zones in red. Heart channel: thick green line shows external pathway; dotted green line shows the internal pathway. Pericardium channel: thick yellow line shows the external pathway; dotted yellow line shows the internal pathway. The acupuncture points with therapeutic indication for heart or lung are highlighted. (b) Posterior view. The acupuncture points with therapeutic indications for heart or lung in the area are highlighted.

The upper area comprises heart and lung zones and corresponds to D1 to D6 dermatomes; in cases of aggravation of the disease, dermatomes D7 to D9 and neck may be included. They relate to clinical cases of cardiac hypertrophy and dilatation, valvulopathy, angina, myocardial infarction, aortic aneurysm, acute pain in pulmonary disease, acute bronchitis, and pain with coughing episodes [14]. This zone presents high variability depending on the involvement of the disease. For this reason the clinical cases referenced by Head's were taken into account for the correlation with acupuncture and its further quantitation. At the anterior side anatomical correlation is observed between this area and the heart channel in chest, upper limb, and infraorbital area. Similarly, it is possible to appreciate the anatomical correlation of this zone with pericardium channel at chest and upper limb (see Figure 2(a)). In posterior thorax and upper limb, anatomical correlation with acupuncture channels was weak but acupuncture points with anatomic and functional correlation with heart and lung physiology were found. Inside the upper area there are also channels of small intestine, lung, San Jiao, and large intestine where anatomical correlation was not so clear.

As shown in Figure 2(a), five channels pass through Head Zones of heart and lung: kidney, stomach, spleen, pericardium, and* Ren*, areas that include 13 acupuncture points. Three channels with 11 acupuncture points were found in the back: bladder, small intestine, and San Jiao. In the upper limb there is correspondence with 13 acupoints of channels of heart, pericardium, and San Jiao. Sir Head did not find any other viscerocutaneous relationships due to the anatomical gap between C5 and C8 [14]. These points show activity on lung, heart, or both (see Table 3).

Similarly, it is observed that the therapeutic activity of acupuncture points can take effect on lung, heart functions, or both. For example,* Ren* 13 and B16 relieve thoracic pain. K 25 clears heat in thorax and chest congestion. B14 alleviates chest pain, cough, and dyspnea (see Table 3) [11, 13, 25]. Functionally, points in small bowel channel are related to heart activity but in a slight manner, because of being paired channels [11, 13, 25].

### 3.3. Middle Area of the Body (Head Zones for Stomach, Liver-Gallbladder and Bowels)

**Figure 3:**
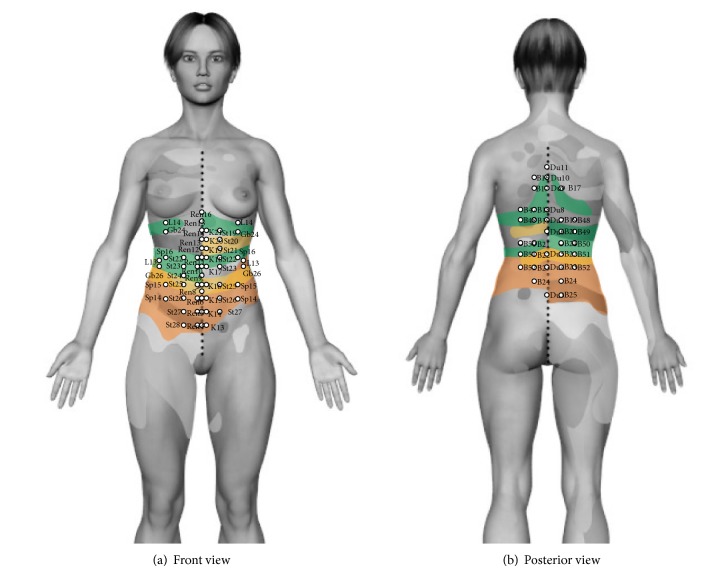
(a) Front view. (b) Posterior view. Head zones of stomach in yellow, zone of liver in green, and zone of gallbladder and bowels zone in orange. The acupuncture points with therapeutic indications in the area are highlighted.

The anatomical correlation with channels is not so evident; however acupuncture points within this area, belonging to different channels, have anatomical and functional correlations (see Figures 3(a) and 3(b) and Table 4).

In Figure 3(a) it is possible to identify three channels of acupuncture in the Head zone of stomach, stomach, kidney, and Ren mai, located between dermatomes D6 to D9 which are related to clinical cases of acute gastritis and gastric ulcer. In addition there are 8 acupoints that belong to these channels. In Figure 5(b), there is correspondence in the back area with two channels: bladder and Du and 5 acupuncture points. Functionally the acupuncture points mentioned above have actions on digestive physiology (see Table 4).

At the anterior Head zone of liver-gallbladder, there are 16 acupoints belonging to 5 channels, gallbladder, kidney, stomach, liver, and* Ren,* matching anatomically when passing through this area (see Figure 3(a) and Table 4). In the back area, Figure 3(b) shows 11 acupuncture points belonging to two channels: bladder and Du. Functionally, these points relate to digestive and metabolic actions of liver in TCM. For instance, the acupoint Gb 24 benefits gallbladder and B18 controls and harmonizes liver [11, 13, 25]. Head found clinical cases associated with liver and biliary colic pain in this area between dermatomes D7 to D10.

In the Head zone of bowel located between dermatomes D9 to D12, rectum from S2 to S4 (see Table 1), there are 15 acupoints belonging to 6 channels: stomach, spleen, kidney, liver, gallbladder, and Ren (see Figure 3(a)). In the back zone, there are 11 acupuncture points belonging to the bladder channels and Du mai (see Figure 3(b)). There are 6 points of the bladder channel in lower limbs, and 2 points in perineal area belonging to Du mai and Ren mai. Functionally this area has intestinal digestive activity. Similarly, points Sp13 to Sp15 of spleen channel regulate lower jiao [11, 13, 25].

There are acupuncture points that perform an effect on more than one zone, such as L13 which has extensive digestive action because of being the Mu point of spleen channel (see Table 4).

In the middle area, there are 74 points related, 39 of them in the region of abdomen belong to 6 channels, 27 in the back belong to two channels, 2 points in perineal area belong to two channels and 6 points in lower limbs belong to one channel.

### 3.4. Lower Area (Head Zone of Kidney-Ureter, Bladder, and Male and Female Genitals)

**Figure 4:**
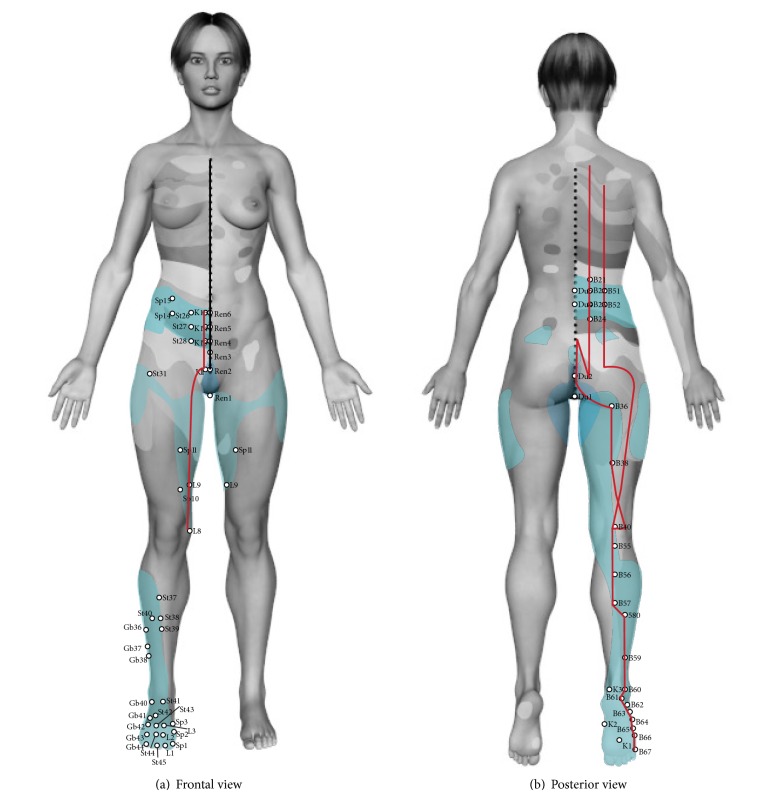
(a) Front view. Correlations between lower limb and lower abdomen with Head zones of kidney, prostate, and uterus with kidney channel highlighted in red. Acupuncture points with therapeutic indication for bladder, kidney-ureter, and genital organs are highlighted. (b) Posterior view. Correlations between lumbosacral and lower limbs area with Head zone of prostate, bladder, and uterus with bladder channel highlighted in red. Acupuncture points with therapeutic indication for bladder, kidney-ureter, and genitalia are highlighted.

Head zone of kidney, prostate, and uterus is located between dermatomes D10 to L1 (see Table 1). As shown in Figure 4(a), there are 8 acupoints in lower abdomen belonging to 4 channels: stomach, kidney, gallbladder, and Ren, anatomically matching when passing through this area. In back area (Figure 4(b)), there are 4 points, belonging to the bladder and Du channels. Seven points belonging to 3 channels, liver, kidney, and spleen, are also observed in lower limb area in the same way that two points are observed in perineal area belonging to* Du mai* and* Ren mai* (Figures 4(a) and 4(b) and Table 5). Functionally, this area is related to urinary activity, as documented in Sir Head clinical findings in patients with renal calculus and renal colic. Similarly, St 27 point benefits and nourishes the kidney and B23 point tonifies kidney functions in TMC [11, 13, 25]. The anatomical correlation between Head zones of kidney, prostate, uterus with kidney channel at the level of lower abdomen, and inner thigh and foot are observable in Figure 4(a).

Head zone of bladder comprises from dermatomes D11 to S4 excluding L2 to L4 due to anatomical gap. In lower abdomen there are 7 acupuncture points belonging to 3 channels: stomach, kidney, and Ren that match anatomically when passing through this area (see Figure 4(a)). In back area there are 4 points belonging to the bladder channel with anatomical correlation. In lower limb there are 4 points belonging to 2 channels: bladder and liver, and finally in perineal area there are two points belonging to* Du mai* and* Ren mai* (Figure 4(b) and Table 5). This area has effects on urinary function, as described by Sir Head in clinical cases of* vesical calculus* and urinary retention. In Figure 4(b), the anatomical correlation is observed between Head zones for prostate, bladder, and uterus with bladder channel at lumbosacral level and the posterior side of thigh and foot.

The genital area is composed of Head zones for prostate, epididymis and annexes, testis and ovary, and cervix and uterus which are located between dermatomes D10 and L5, excluding L2 to L4 because of anatomical gap (see Table 1). In the lower abdomen there are 13 acupuncture points belonging to four channels: stomach, kidney, gallbladder, and Ren, anatomically matching when passing through this area (see Figure 4(a)). At the back area there are 5 points from two channels: bladder and Du. In lower limbs there are 8 points belonging to 2 channels: bladder and liver. In perineal area there are 2 points belonging to 2 channels, Ren and Du (Figure 4(b) and Table 5). The actions on genital and reproductive functions are related to the clinical descriptions of genital infections, and malignant diseases by Head. In TCM, Du2 acupoint benefits the lumbar area and alleviates leucorrhoea and irregular menstruation [11, 13, 25].

In lower area 79 total acupoints were found: 28 of them in the low abdomen belonging to 4 channels, 13 acupoints in the back belonging to 2 channels, 32 acupoints in lower limb from 2 channels, and 6 acupoints in the perineal region corresponding to 2 channels.

## 4. Discussion

This paper is a research in the field of neural therapy, looking for a descriptive and comparative analysis to identify the anatomical and functional correlations between channels and acupoints in TCM and the viscerocutaneous relationships found by Head as a contribution for neural therapy practice.

**Figure 5:**
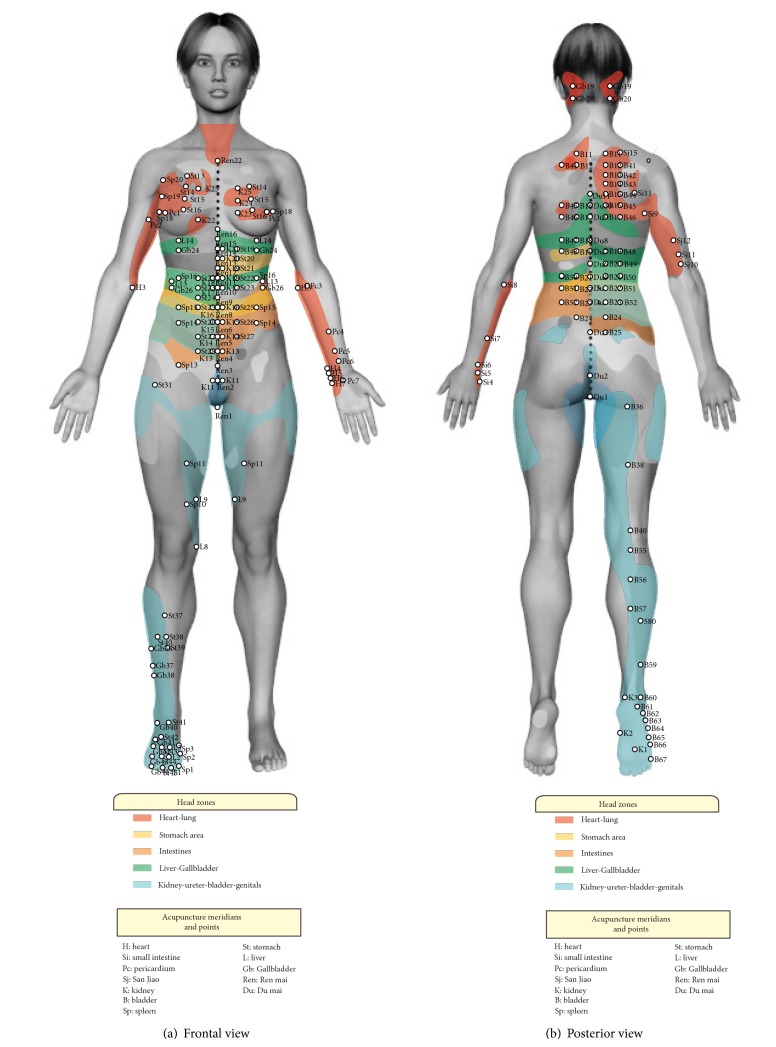
(a) Front view. (b) Posterior view. Head zones in color: heart and lung zones in red; stomach zone in yellow; bowel zone in orange; liver and gallbladder zones in green; ureter-kidney, bladder, and genitalia zones in blue. White dots: acupuncture points with similar functional activity at Head zone.

235 acupoints share anatomical and functional correlation with Head zones. The greater anatomical and functional correlation between channels and head zones corresponds to channels of heart, pericardium (Figure 2(a)), and kidney and bladder (Figures 4(a) and 4(b)). Anatomo-functional relationships found with other channels were not remarkable. Acupoints through channel of bladder in back exhibit functional-segmental relationship with the organs where it goes through, as documented by other studies (Figures 4(b) and 5) [21, 22, 26].

Several Head zones of head and neck show anatomic and functional relations with acupoints because of their remote action on thoracic-abdominal organs. Analyzing the description of Head zones of stomach, liver, gallbladder, and bowels, a partial overlap of these three areas is evident. Equivalently, in TCM, there are acupuncture points that share therapeutic indications. A similar overlap occurs between Head zones of kidney, bladder, and sexual organs with acupoints located within this area due to having simultaneous activity in the 3 zones. These overlapped areas and functions can be explained by the configuration on the nervous system of the abdominal visceral information, which is collected by the autonomous nervous system through afferents from the sympathetic nervous system and vagus nerve. These afferences pass through celiac ganglion and mesenteric ganglions and go to the dorsal horn of spinal cord where the information is integrated with dermatomes [1, 17, 19]. Furthermore, urinary and genital systems have a common embryological origin from the intermediate mesoderm [17]. In accordance with this finding, TCM considers that bladder and kidney are coupled organs, related to the regulation of urinary, sexual, and reproductive physiology, among other functions [12, 13]. This anatomical and embryological correlation between acupuncture channels and Head zones is also functionally related to acupuncture points, reinforcing the theory of viscerocutaneous reflexes [27–29].

Recent authors have studied anatomical and physiological relationships between channels and acupuncture points with the embryological metameres, including the exploration Head zones [30]. In other studies the production and expression of somatic pain is compared with visceral pain, finding quantitative, qualitative, chemical, and anatomical differences in both as well as in afferent innervation density, type of innervation, and processing mechanisms and receptors involved [28, 29, 31]. There are studies about the viscero-somatic responses that ascend through the dorsal column of the spinal cord and connecting with spinothalamic tract. It seems that the information from segment level integrates with efferents of somatic origin and produces hyperalgesia, hypothermia, dystrophy, and pressure pain phenomena described by Head [32–34]. These responses also stimulate neuropeptides and local and remote nociceptors subcutaneously. The ability of the extracellular matrix to act as a mechanical network, is being evaluated, in which, the physiological activities of the cells, interstitial elements and signals interact through the whole body [35, 36]. This is consistent with Pischinger and Heine's Basic System Theory, when proposing that the extracellular matrix along with other cellular, humoral, and nervous elements is distributed throughout the body and constitutes a regulatory system responsible for communication, defense, and adaptation [37–39].

It is worth highlighting that channels and acupuncture points as well as Head zones come from the clinical observation of patients with changes in tenderness, pain, subcutaneous skin stiffness, and cephalea associated with organic diseases [30]. Although the clinical study of Head is much more recent and less complex than the compendium of TCM, it is interesting to realize the viscerocutaneous relationships they share, which are not considered enough in allopathic medicine. On the other hand, Sir Head mentioned that there are painful points of variable locations that can be found beyond the areas described, points related to visceral pathology [14]. In a similar way TCM describes* ashi* points of variable locations outside the channels, which are painful on palpation [11].

These results could provide a support for many of the segmental and remote reactions which are evident in the daily practice of neural therapy, through the concepts of* interference field* or* neurodystrophy*, as well as providing to the neural therapist a broad spectrum of knowledge about possible responses to specific therapeutic interventions.

Even though Sir Head describes within his findings on head and neck a relation of tenderness in face associated with dental disease, these findings are not treated in the present work but they could be considered for future research. There was no discussion about the “maximum points” described by Head because they were already been treated in another work (2011) [20].

Head zones have changed over time in terms of segmentation of dermatomes [15], a reason to develop more accurate searching and evaluation methods. This work can also provide acupuncture explanations of traditional concepts, through anatomical, physiological, embryologic, and metameric foundations.

It is relevant to highlight that the findings of this research ought to be used under the neural therapy individuality principles [3] because it is not possible to establish the same protocol for all patients with visceral disease.

## 5. Conclusions

There are clear anatomical and functional associations between Head zones, channels, and acupuncture points. These associations have the viscerocutaneous reflexes as common axis. Similarly, remote relationships may have their origin in the biological information flow that occurs through the basic substance. This hypothesis has been supported in recent studies. However, in areas where there is no obvious anatomical or functional correlation, it cannot be ruled out that there are other biological communication paths beyond this analysis. The finding of these correlations may help in future anatomical, embryological, and clinical investigation in the field of neural therapy and TCM.

## Figures and Tables

**Table 1:** Relation of Head Zones with dermatomes in torso and head and neck areas, according to description of clinical cases for each zone. Dermatome varies according to disease aggravation (in bold).

Head zone	Related dermatome	Areas on face and neck
Heart (H) and lung (L) (1)	H: D1–D3, **D4–D9** and cervical L: D1–D5, **D6-D7**	Frontonasal Frontotemporal Midorbital

Stomach	D6–D9	Frontonasal Frontotemporal Temporal Parietal vertical

Liver (LV) and gallbladder (G)	L: D7–D10 Right side GB: D7–D9. **D6**	Frontonasal Vertical occipital (very common in neck and shoulder pain)

Bowel	(a) Until rectum: D9–D12 (b) Rectum: S2–S4	Vertical Parietal

Kidney and ureter	D10 to L1	No relationship is described

Bladder	(a) Overdistension: D11, D12, L1 (b) Membrane and neck: **S1**, S2–S4	No relationship is described

Sexual organs (2)	D10–S4, L5. Excluding L2 to L4 by anatomical gap	Occipital (3)

(1) The areas of heart and lung share dermatomes, as well as areas in face, which were unified in this work for practical purposes of analysis. (2) The author shows a different dermatome for each sexual organ: prostate (D10, D11 D12, L5, S1–S3), epididymis and annexes (D11-D12, L1, D11-D12, L1), testis and ovary (D10), cervix and uterus ((a) in contraction: D10–D12, L1, (b) cervical canal and lower portion. S1, S2–S4, L5). They were unified for purposes of analysis. (3) Only described for ovarian and testicular dermatome.

**Table 2:** Relationships between Head zone of head and neck with organs at local and remote way, with dermatomes in torso and acupuncture points in head and neck.

Head and neck Head's zones	Related organs	Related dermatomes in torso	Acupuncture points in head and neck
Frontonasal	LA: eye, nose, and upper teeth RA: heart, lungs, stomach, and liver	C3-C4	LA: *Du*24 RA: B2, B3, and B4

Mid-orbital	LA: eye RA: lung and heart	D2, D3, D4	LA: E1, B5, Gb14, Gb15, and Du23. RA: Not found

Frontotemporal	LA: eye RA: lung and heart	D4, D5, D6	LA: Sj23, Gb1, and Gb13 RA: Not found

Temporal	LA: eye and upper teeth RA: Lung, heart, and stomach	D7	LA: Not found RA: Not found

Vertical	LA: eye and ear RA: stomach, liver, and small intestine	D8	LA: Gb16, Gb18, Gb4, Gb5, and Gb6 RA: B6, Gb17, and St8

Parietal	LA: ear RA: stomach and small intestine	D9	LA: Sj20, and *Du*20 RA: B8, Sj19, Gb3, Gb7, Gb8, and Gb9

Occipital	LA: occipital and dorsal RA: liver, bowels, ovary, and testis	D10	LA: B9, Gb10, Gb19, *Du*17, and *Du*18 RA: Not found

Sternomastoid and sternonuchal	LA: not described RA: lung and heart	C3-C4	LA: B10, Gb20, and *Du1*5 RA: St11 to St15, Gb21, *Du*16, Li17, and Li18

RA: Remote action. LA: local action.

**Table 3:** Acupoints related to anatomical and functional zones for heart and lung.

Head zones	Acupuncture points	Total acupoints (acp) and channels (ch) related
Heart and lung	Chest K23 to K25, St13 to St16, Sp18 to Sp20, *Ren*22, Pc1, and K22	13 acp 5 ch
	Back Si11, Si15, B11 to B13, B42, B45, B15, B16, Sj15, and B14	11 acp 3 ch
	Upper limb H1, H3 to H7, Pc2 to Pc7, and Sj10	13 acp 3 ch

Total		37 acp

**Table 4:** Acupuncture points, anatomo-functionally related to stomach, liver, gallbladder, and bowels Head zones.

Head zones	Acupuncture points
Stomach	Abdomen: St20, St21, St25, K16, K19, K20, *Ren*12, and *Ren*13 Back: B19, B21, B22, B48, and B51

Liver-Gallbladder	Abdomen: St19, St22–St24, Sp16, L13, L14, Gb24, Gb26, K17, *Ren*9 to *Ren*11, and *Ren*14 to *Ren*16 Back: B16 to B18, B21, B22, B47, B50, and *Du*6 to *Du*9

Bowels	Abdomen: St26, St27, St28, Sp13 to Sp15, K4, K13 to K15, L13, *Ren*4, *Ren*5, *Ren*6, and Gb25 Back: B21, B22 to B25, B33, B34, B35, B52, *Du*4, and *Du*5 Perineal: *Du*1, *Ren*1 Lower limb: B36, B37, B38, B40, B56, B57

**Table 5:** Acupoints anatomo-functionally related to Head zones for kidney, bladder and genitalia.

Head zones	Acupuncture points
Kidney	Lower abdomen: St27, K13, Gb25, and *Ren*2 to *Ren*6 Back: B23, B52, B53, and *Du*4 Perineal: *Du*1 and *Ren*1 Lower limb: H1, Sp10, Sp11, L8, L9, K1, and K3

Bladder	Lower abdomen: St27, St28, K11, and *Ren*2 to *Ren*5 Back: B22, B23, B52, and B53 Perineal: *Du*1 and *Ren*1 Lower limb: B67 and L1 to L3

Genitalia	Lower abdomen: St26, St28, K11, K13 to K15, Gb27, Gb28, and *Ren*2 to *Ren*6 Back: B23, B24, B52, *Du*2, and *Du*4 Perineal: *Du*1 and *Ren*1 Lower limb: B36, B40, B55, B56, B60, B65, B67, L1 to L3, L8 to L11, Gb44, Sp1, Sp10, Sp11, and K1 to K3
